# Developmental responses to lung injury: repair or fibrosis

**DOI:** 10.1186/1755-1536-5-S1-S2

**Published:** 2012-06-06

**Authors:** David Warburton

**Affiliations:** 1 Developmental Biology and Regenerative Medicine Program, Saban Research Institute, Children's Hospital Los Angeles, Keck School of Medicine and Ostrow School of Dentistry, University of Southern California, 4650 Sunset Boulevard, Los Angeles, California, 90027, USA

## Abstract

Lung development is a complex and finely balanced process. Yet the lung has a relatively limited repertoire of responses to injury, which, depending on severity of the injury and developmental stage and susceptibility of the lung, culminate in stopping development, followed by more or less successful repair or alternatively in fibrosis. Unlike fetal skin, which heals scarlessly early in gestation, but scars later in gestation and increasingly so postnatally, the damaged fetal lung does heal, but not very well. Thus lung injury appears to entrain a default developmental/repair mechanism involving increased amounts of activated TGF beta ligand signaling. When this occurs prior to or very early in the process of alveolarization, excessive TGF beta ligand inhibits further alveolarization, a disease process phenotype that has been termed Bronchopulmonary Dysplasia in extreme human prematurity. However, once alveolarization is sufficiently advanced as in mid to late gestation fetal monkey, late gestation human or adult mouse, rat or human lung, excessive TGF beta signaling results in pulmonary fibrosis. Recently we have further shown that FGF10 signaling, a process that is necessary for distal lung morphogenesis, can also antagonize bleomycin-induced lung fibrosis in adult mice by a mechanism involving inhibition of active TGF beta ligand bioavailability. We therefore suggest that lung development, repair and fibrosis have many fundamental mechanisms in common, that potentially can be manipulated using cells or soluble factors that optimize the alveolar milieu to prevent and possibly even to reverse lung fibrosis.

## 

Lung development is a complex and finely balanced process of self assembly that is driven by crosstalk between the epithelium, the mesenchyme, the vasculature within it, smooth muscle, nerves and other crtical tissue components including matrix proteins and proteoglycans, together with carefully calibrated responses to physical forces including luminal hydraulic peristalsis [1,2]. Yet the lung has a relatively limited repertoire of responses to injury, which, depending on severity of the injury and developmental stage and susceptibility of the lung, culminate in stopping development, followed by more or less successful repair or alternatively in fibrosis.

In extremely premature human infants (<28 weeks gestation), alveolarization has barely begun. Removal of airway liquid and exposure of the lung to air or oxygen breathing, even with artificial surfactant therapy frequently results in a form of alveolar hypoplasia termed "new" Bronchopulmonary dysplasia (BPD). Whereas before the advent of surfactant therapy, delivery at >30 weeks and ventilation with oxygen to treat acute respiratory distress syndrome resulted in a more classical form of BPD characterized by emphysema and interstitial fibrosis. Long-term follow up of human prematures has shown that their lungs do grow. However, in severe cases of both old and the more newly recognized forms BPD, chronic desaturation requiring home oxygen or even home ventilation is a continuing management issue. Nevertheless in milder cases of prematurity, lung development appears to become relatively normal, with the notable exception that children identified with BPD who survive over 5 years of age destaurate on maximal exercise [3], and there is a markedly increased incidence of wheezing in these children as well. Moreover, their pulmonary reserve in the face of intercurrent viral infection is clearly limited. Thus, unlike the fetal skin, which heals scarlessly early in gestation, but scars later in gestation, and increasingly so postnatally, the damaged human fetal lung does appear to heal, but not necessarily do so very well. In fact a few of the early, larger premature babies who survived Wilson-Mikity syndrome, a BPD-like lung disease, are now showing up at 30 years plus follow up with severe early onset emphysema/COPD like pictures on chest radiography. Of course some of them may be mutant for one of the rare alleles that cause surfactant protein deficiency or misfolding, which themselves result in chronic alveolar epithelial endoplasmic reticulum stress syndromes [4].

Postnatally the lung continues to grow, adding alveolar complexity to achieve the final ventilation-perfusion surface area of a tennis court by young adulthood. However, past middle age, FEV1 falls inexorably by 2-3% per year so that by 120 years of age even super athletes will have COPD/senile emphysema-like restrictive lung disease [5,6].

The impact of lung injury on lung function has been studied quite extensively in animals. In mice and rats, in which alveoalrization occurs postnatally, exposure to hyperoxia or to advector expressing active TGFbeta1 ligand results in a similar BPD-like picture of alveolar hypolplasia [7]. Interestingly though, in fetal monkeys wherein alveolarization is already well advanced in utero, injection of advector expressing active TGFbeta1 ligand results in aggressive fibrosis that extends through the lung, probably via the interstitial reticulum, as far as the visceral pleura, wherein it causes extensive pleural thickening [8]. This phenotype is very similar to the aggressive interstitial fibrosis caused by injection or inhalation of advector expressing active TGFbeta1 ligand in adult rats [9]. However severe pleural thickening is not common in human BPD.

We as well as others others have shown previously that TGF beta signaling plays a key physiological role in lung development as well as alveolar epithelial repair [10]. Therefore lung injury appears to entrain a default repair mechanism involving increased amounts of activated TGF beta ligand signaling. When this occurs prior to or very early in the process of alveolarization, excessive TGF beta signaling inhibits further alveolarization, a disease process phenotype strikingly similar to the one that has been termed Bronchopulmonary Dysplasia in extreme human prematurity. However, once alveolarization is sufficiently advanced as in fetal monkey, or in late gestation human or adult mouse, rat or human lung, excessive TGF beta signaling results in pulmonary fibrosis. This, insight led us to coin the "Goldilocks" hypothesis: that TGF beta signaling has to be "just right" for lung development to function correctly prenatally as well as postnatally.

Interestingly, TGF beta1 ligand signaling also has an important role in suppressing excessive tissue inflammation, as demonstrated in the TGFbeta1 null mouse, which dies of pneumonia or excessive inflammation of other organs. Somewhat similarly null mutation of Smad3 a key downstream signal transduction element in the canonical TGF beta pathway impairs alveolarization, giving rise to a primary hypo-alveolar phenotype, together with subsequent activation of MMP9, which leads to an early onset destructive emphysema phenotype [11,12]. These forms of emphysema are exacerbated by exposure of young mutant mice to sidestream tobacco smoke. Other mutations in TGF beta binding protein genes such as LTBP4, endoglyn or fibrillin result in lung epithelial or vascular malformations.

Probably because they contain intact genomes and are therefore smart, stem cell based therapies are beginning to show promise as methods to ameliorate lung injury and possibly fibrosis. However, the danger that the abnormal fibrotic tissue milieu may induce them to express collagen and other pro-fibrotic proteins remains at least a theoretical concern. Nevertheless stem cells derived from discarded fetal materials may have some theoretical advantages. We have enjoyed some preliminary success using human amniotic fluid derived stem and progenitor cells to ameliorate the tissue milieu in various injury models, including the lung and the kidney [13].

Gremlin is an intracellular as well as extracellular BMP inhibitor ligand binding protein that inhibits the function of BMP4 in the developing lung [14]. Interestingly, Gremlin is over-expressed in cases of adult idiopathic pulmonary fibrosis (IPF) [15], its level of expression can differentiate among severities of IPF and it does indeed antagonize BMP signaling and thus potentiates pulmonary fibrosis [16,17].

We have also recently shown that FGF10 signaling, a process that is necessary for distal lung morphogenesis, can also antagonize bleomycin-induced lung fibrosis in adult mice, by a mechanism involving inhibition of active TGF beta ligand bioavailability and hence less tissue inflammation [18]. This suggests that modification of the alveolar milieu may also be possible, using soluble developmental factors that could ameliorate or reverse TGFbeta mediated fibrosis.

## Competing interests

The authors declare that they have no competing interests.

## References

[B1] Warburton D El-Hashash A Carraro G Tiozzo C Sala F Rogers O De Langhe S Kemp PJ Riccardi D Torday J Bellusci S Shi W Lubkin SR Jesudason E Lung organogenesis Curr Top Dev Biol 2010 90 73 158 20691848 10.1016/S0070-2153(10)90003-3 PMC3340128

[B2] Jesudason EC Keshet E Warburton D Entrained pulmonary clocks: epithelium and vasculature keeping pace Am J Physiol Lung Cell Mol Physiol 2010 299 4 L453 L454 10.1152/ajplung.00263.2010 20693313

[B3] Bader D Ramos AD Lew CD Platzker AC Stabile MW Keens TG Childhood sequelae of infant lung disease: exercise and pulmonary function abnormalities after bronchopulmonary dysplasia J Pediatr 1987 110 5 693 699 10.1016/S0022-3476(87)80004-5 3572620

[B4] Korfei M Ruppert C Mahavadi P Henneke I Markart P Koch M Lang G Fink L Bohle RM Seeger W Weaver TE Guenther A Epithelial endoplasmic reticulum stress and apoptosis in sporadic idiopathic pulmonary fibrosis Am J Respir Crit Care Med 2008 178 8 838 846 10.1164/rccm.200802-313OC 18635891 PMC2566794

[B5] Fletcher C Peto R The natural history of chronic airflow obstruction Br Med J 1977 1 1645 1648 10.1136/bmj.1.6077.1645 871704 PMC1607732

[B6] Shi W Warburton D Is COPD in adulthood really so far removed from early development? Eur Respir J 2010 35 1 12 13 10.1183/09031936.00145809 20044457

[B7] Gauldie J Galt T Bonniaud P Robbins C Kelly M Warburton D Transfer of the active form of transforming growth factor-beta 1 gene to newborn rat lung induces changes consistent with bronchopulmonary dysplasia Am J Pathol 2003 163 6 2575 2584 10.1016/S0002-9440(10)63612-7 14633629 PMC3278797

[B8] Tarantal AF Chen H Shi TT Lu CH Fang AB Buckley S Kolb M Gauldie J Warburton D Shi W Overexpression of transforming growth factor-beta1 in fetal monkey lung results in prenatal pulmonary fibrosis Eur Respir J 2010 36 4 907 914 10.1183/09031936.00011810 20351039 PMC3074616

[B9] Sime PJ Xing Z Graham FL Csaky KG Gauldie J Adenovector-mediated gene transfer of active transforming growth factor-beta1 induces prolonged severe fibrosis in rat lung J Clin Invest 1997 100 4 768 776 10.1172/JCI119590 9259574 PMC508247

[B10] Buckley S Shi W Barsky L Warburton D TGF-beta signaling promotes survival and repair in rat alveolar epithelial type 2 cells during recovery after hyperoxic injury Am J Physiol Lung Cell Mol Physiol 2008 294 L739 L748 10.1152/ajplung.00294.2007 18245268

[B11] Chen H Sun J Buckley S Abnormal mouse lung alveolarization caused by Smad3 deficiency is a developmental antecedent of centrilobular emphysema Am J Physiol Lung Cell Mol Physiol 2005 288 L683 L691 15591413 10.1152/ajplung.00298.2004

[B12] Chen H Zhuang F Liu YH TGF-{beta} receptor II in epithelia versus mesenchyme plays distinct role in developing lung Eur Respir J 2008 32 285 295 10.1183/09031936.00165407 18321928 PMC2865234

[B13] Perin L Sedrakyan S Giuliani S Da Sacco S Carraro G Shiri L Lemley KV Rosol M Wu S Atala A Warburton D De Filippo RE Protective effect of human amniotic fluid stem cells in an immunodeficient mouse model of acute tubular necrosis PLoS One 2010 5 2 e9357 10.1371/journal.pone.0009357 20195358 PMC2827539

[B14] Shi W Zhao J Anderson KD Warburton D Gremlin negatively modulates BMP-4 induction of embryonic mouse lung branching morphogenesis Am J Physiol Lung Cell Mol Physiol 2001 280 5 L1030 1039 11290528 10.1152/ajplung.2001.280.5.L1030

[B15] Koli K Myllärniemi M Vuorinen K Salmenkivi K Ryynänen MJ Kinnula VL Keski-Oja J Bone morphogenetic protein-4 inhibitor gremlin is overexpressed in idiopathic pulmonary fibrosis Am J Pathol 2006 169 1 61 71 10.2353/ajpath.2006.051263 16816361 PMC1698771

[B16] Myllärniemi M Vuorinen K Pulkkinen V Kankaanranta H Aine T Salmenkivi K Keski-Oja J Koli K Kinnula V Gremlin localization and expression levels partially differentiate idiopathic interstitial pneumonia severity and subtype J Pathol 2008 214 4 456 463 18072275 10.1002/path.2300

[B17] Myllärniemi M Lindholm P Ryynänen MJ Kliment CR Salmenkivi K Keski-Oja J Kinnula VL Oury TD Koli K Gremlin-mediated decrease in bone morphogenetic protein signaling promotes pulmonary fibrosis Am J Respir Crit Care Med 2008 177 3 321 329 17975199 10.1164/rccm.200706-945OC PMC2218851

[B18] Gupte VV Ramasamy SK Reddy R Lee J Weinreb PH Violette SM Guenther A Warburton D Driscoll B Minoo P Bellusci S Overexpression of fibroblast growth factor-10 during both inflammatory and fibrotic phases attenuates bleomycin-induced pulmonary fibrosis in mice Am J Respir Crit Care Med 2009 180 5 424 436 10.1164/rccm.200811-1794OC 19498056 PMC2742760

